# Investigating the impact of captivity and domestication on limb bone cortical morphology: an experimental approach using a wild boar model

**DOI:** 10.1038/s41598-020-75496-6

**Published:** 2020-11-04

**Authors:** Hugo Harbers, Clement Zanolli, Marine Cazenave, Jean-Christophe Theil, Katia Ortiz, Barbara Blanc, Yann Locatelli, Renate Schafberg, Francois Lecompte, Isabelle Baly, Flavie Laurens, Cécile Callou, Anthony Herrel, Laurent Puymerail, Thomas Cucchi

**Affiliations:** 1 Archéozoologie, Archéobotanique: Sociétés, Pratiques et Environnements, UMR 7209, Muséum national d’Histoire naturelle, CNRS, Paris, France; 2 Laboratoire PACEA, UMR 5199, Université de Bordeaux, Bordeaux, France; 3 School of Anthropology and Conservation, Skeletal Biology Research Centre, University of Kent, Marlowe Building, Canterbury, Kent, CT2 7NR UK; 4 Department of Anatomy and Histology, School of Medicine, Sefako Makgatho Health Sciences University, Pretoria, South Africa; 5 Mécanismes Adaptatifs et Evolution, UMR 7109, Muséum national d’Histoire naturelle CNRS, Paris, France; 6 Réserve Zoologique de la Haute Touche, Muséum national d’Histoire naturelle, Obterre, France; 7 Physiologie de la Reproduction et des Comportements, UMR 7247, National Research Institute for Agriculture, Food and Environment (INRAE), CNRS Université de Tours IFCE, Nouzilly, France; 8 Central Natural Science Collections, Martin Luther University Halle-Wittenberg, Halle, Germany; 9 Plateforme CIRE, National Research Institute for Agriculture, Food and Environment (INRAE), Nouzilly, France; 10 Unité Bases de Données sur la Biodiversité, Écologie, Environnement et Sociétés, UMS 3468, Muséum national d’Histoire naturelle, Paris, France; 11 Anthropologie bio-culturelle, droit, éthique et santé (ADES), UMR 7268, Faculté de Médecine Site Nord, Marseille, France

**Keywords:** Ecology, Evolution, Zoology, Ecology, Anatomy

## Abstract

The lack of bone morphological markers associated with the human control of wild animals has prevented the documentation of incipient animal domestication in archaeology. Here, we assess whether direct environmental changes (i.e. mobility reduction) could immediately affect ontogenetic changes in long bone structure, providing a skeletal marker of early domestication. We relied on a wild boar experimental model, analysing 24 wild-born specimens raised in captivity from 6 months to 2 years old. The shaft cortical thickness of their humerus was measured using a 3D morphometric mapping approach and compared with 23 free-ranging wild boars and 22 pigs from different breeds, taking into account sex, mass and muscle force differences. In wild boars we found that captivity induced an increase in cortical bone volume and muscle force, and a topographic change of cortical thickness associated with muscular expression along a phenotypic trajectory that differed from the divergence induced by selective breeding. These results provide an experimental proof of concept that changes in locomotor behaviour and selective breeding might be inferred from long bones morphology in the fossil and archaeological record. These trends need to be explored in the archaeological record and further studies are required to explore the developmental changes behind these plastic responses.

## Introduction

Exploring the process of domestication as an integration of animals into human society provides a unique insight into one of the key steps in *Homo sapiens* evolution, at the root of its global impact over the biosphere^1^ and species evolution^2^. However, documenting this process, as an intensification of the relationship between humans and animals in archaeology^3^, is challenging^4,5^. One of the main issues is that no relevant methodological approach has been able to capture this elusive process. Bioarchaeologists have relied on a morphological ‘syndrome of domestication’ of the skeleton first proposed by Darwin^6^ and later tested experimentally by Belyaev in his famous fox farm experiment^7–9^. These morphological syndromes, including bone size reduction and changes in craniofacial morphology, have often been considered as a pleiotropic consequence of tameness selection^10^ through the perturbation of the neural crest cells involved in bone and chondral development^11^. However, these syndromes imply complete genetic isolation and strong artificial selection, which are not transferable to the early process of domestication by the first farming communities^12,13^. Furthermore, syndromes are not shared across species and mainly relate to breeding rather than behavioural selection^14^. For all these reasons, new morphological markers are required to further document the early interaction between the ecological dynamics of humans and animals based on archaeological remains.

Here, we provide an experimental proof of concept that direct environmental changes (i.e. mobility reduction) could immediately affect ontogenetic changes in long bone structure, providing a skeletal marker of early domestication. One of the least understood aspects when documenting animal domestication in archaeology is the morphological response to environmental conditions experienced by animals under human control^5^. To date, it has been generally considered that morphological changes such as bone size reduction or craniofacial modifications are subsequent to the integration of animals into human society^15,16^. Therefore, morphological markers have been deemed irrelevant to document the initial domestication process as they would only be detectable in an already domesticated animal; i.e. once genetic isolation and breeding selection were already in place^3^. However, the ecological responses to the environmental stress of human control at an individual scale may induce phenotypic plasticity that can be quantified by its reaction norm^17^. Phenotypic plasticity is the outcome of the interaction between the genotype and the environment through development without any genetic mutations^18^. The growth of bones is directly affected by their habitual loading environment^19^. Activity and motion will produce muscular strains and loading that bones will have to resist. The bone resistance to these stresses can be achieved through bone mass, bone geometry and reorganization of bone microstructure with modelling activity^20^. The bone adaptation to mechanical stimuli during growth is immediate and can last into adulthood. So far, the plastic response of animal’s bone morphology under human control has been be investigated in archaeology through bone pathologies^21^ and discrete morphological differences (see refs. in^5^). Histological differences have been used to document variations between wild specimens and domestic breeds ^22,23^. Despite the well-known plasticity of limb bones to changes in the biomechanical stimuli of their environment through bone growth (modelling) and turnover (Haversian remodelling) of their shaft^20^, no studies have used this framework to explore how human control of the natural behaviour of a wild animal impacts its limb bone structure and how these changes might inform the early process of animal domestication.

The ability of the limb bone to adapt its mass, shape and architecture to fit the biomechanical demands that prevail in its environment has been recently investigated in rodents^24^ and mustelid^25–27^ mammals. Since cortical bone is well preserved in fossil and archaeological deposits, researchers in physical anthropology have investigated structural variation in limb bones in relation to environmental variation to infer locomotor adaptations and behaviour in past hominins. These approaches include the study of the diaphyseal cross-sectional geometry^20,28^ or markers of muscle insertions visible on the periosteal surface, also known as entheseal changes^29–32^. More recently, virtual imaging has allowed the development of a comprehensive approach to study variation in cortical thickness in limb bones using 3D morphometric mapping^33–39^. However, Pearson and Lieberman^20^ argued for caution in the interpretation of these markers to reconstruct past human behaviour without further experimental approaches. Subsequent studies explored the relevance of functional inferences from bone morphology using experimental studies on domestic vertebrates^40–42^ or cross-sectional studies on primates^37^. These studies supported the validity of the reconstruction of locomotor behaviour based on changes in the cortical structure of limb bones. Yet, no support was found for a relation between entheseal changes and variation in locomotor behaviour. Experiments using electrical muscle stimulation in mice, however, show that entheseal changes can reflect the repetitive use of muscles^43^.

This study tested experimentally the hypothesis that reduced mobility during growth in a wild ungulate, induced by captivity, results in measurable structural variation in limb bone morphology. Considering that physical activity stimulates bone remodelling, we expected that a free-ranging wild ungulate, having space to fully express their locomotor behaviour, to have a thicker cortical bone in the humerus than a wild ungulate which had grown in captivity. If such plastic responses could be quantitatively differentiated from the reaction norm in extant wild ungulates exhibiting natural behaviour, it would represent a marker of human control over wild animal movements, transferable to the archaeological record in order to reconstruct the early process of human control of wild animal populations^44^. This study relied on a large-scale experiment controlling genetic and environmental factors while imposing changes in locomotor behaviour during growth in a population of captive wild boar (*Sus scrofa*). The structural changes in the limb bones were quantified using 3D morphometric mapping of cortical thickness. We evaluated the effects of age, body mass, sex, and muscle cross-sectional area. The captivity signal in the bone morphology of the experimental wild boars was contrasted with the cortical signal from free-ranging wild boar populations, to explore how much the response to captivity can be differentiated from the reaction norm of bones of animals in their natural habitat. Finally, we contrasted the captivity signature with the impact of selective breeding on bone cortical morphology using pigs from traditional and industrial breeds to assess whether breeding selection and mobility control can be distinguished from one another.

## Material and methods

### Experimental design

To test how a change in locomotor behaviour through mobility reduction affects the topographic variation of the cortical thickness of the humerus shaft in a wild ungulate, we relied on a genetically homogenous population of wild boar living in a 100,000 m^2^ (10 ha) fenced forest in Urciers (Indre, France) thus controlling for variation in genetic diversity and environment. Human interaction with this population was intentionally kept to a minimum, ensuring that the behaviour of the boars remained as natural as possible. From this controlled population we captured 24 6-month-old piglets that we divided into two groups of equal sample size and sex ratio. Both groups were raised until the age of 24 months under two different contexts of mobility: a 3000 m^2^ (0.3 ha) wooded pen and an indoor stall of 100 m^2^, where males and females were separated. We created two captive settings, expecting that they would induce a difference in the amount of movement , sufficient to produce observable changes in humerus cortical bone. Both groups were supplied with standardized food pellets in order to maintain a healthy weight, according to the standard nutritional requirements of European wild boar populations (Étienne 2003). Water was available ad libitum.

This experiment was undertaken in the zoological reserve of La Haute Touche, Obterre (France) and received full ethical agreement from the Ethical Committee for animal experimentation of the Natural History Museum of Paris (Comité Cuvier) and the French Ministry of higher education and research (APAFIS#5353-201605111133847). This experiment was performed in accordance with relevant guidelines and regulations.

### Comparative collection

The captivity signal in the experimental wild boars at adulthood was contrasted with the variation in morphology observed across 23 wild-caught adult wild boars from the control population in Urciers (Indre, France) and three other populations in France (Table 1). All these specimens were wild caught between 1 and 3 years of age.

**Table 1:** Sample origin and number of available specimens for the different parameters analysed in this study.

Status	Category	Population/breed	Mobility	Curation	N cartography	N muscle data	N body mass	Grouping factor
Wild boar	Control (France)	Urciers	Wild caught	MNHN	5	1	5	WB_ctrl
Wild boar	Experiment (France)	Urciers	Captive reared (stall)	MNHN	12	10	12	WB_stall
Wild boar	Experiment (France)	Urciers	Captive reared (pen)	MNHN	12	12	12	WB_pen
Wild boar	France	Compiègne	Wild caught	MNHN	4	0	4	WB_wc
Wild boar	France	Chambord	Wild caught	MNHN	14	6	11	WB_wc
Pigs	Landraces	Bayerisches Landschwein	Captive reared (stall)	MHK	5	0	0	PIG_Land
Pigs	Landraces	Hannover-Braunschweig Landschwein	Captive reared (stall)	MHK	5	0	0	PIG_Land
Pigs	Landraces	Mangalitza	Captive reared (stall)	MHK	1	0	0	PIG_Land
Pigs	Landraces	Polnisches Landschwein	Captive reared (stall)	MHK	1	0	0	PIG_Land
Pigs	Landraces	Corsican Breed	Free range	MNHN	5	0	0	PIG_Cor
Pigs	Improved breeds	Berkshire	Captive reared (stall)	MHK	4	0	0	PIG_Improv
Pigs	Improved breeds	Unknown	Captive reared (stall)	MNHN	1	0	0	PIG_Improv

*MNHN* Muséum national d’Histoire naturelle in Paris, *MHK* Museum für Haustierkunde Julius Kühn in Halle, *MHNG* Muséum d’Histoire Naturelle in Geneva. For information regarding body mass, age, sex, muscles and status of the individuals included, please see data availability.

To compare the plastic response of captivity with the phenotypic change induced by the last 200 years of artificial selection, we collected data on 17 domestic pigs, including 12 specimens from traditional landraces, which were part of a conservation programme, and five from an intensive breeding programme dedicated to industrial meat production (Table 1). All the domestic specimens are part of the historical collections of the MHK (*Museum für Haustierkunde Julius Kühn*, Halle). They were reared in stalls and were aged between 1 and 5 years of age. We also included five free-range Corsican landrace pigs (*U nustrale*) aged between 14 and 18 months. These pigs were bred according to traditional and extensive herding practices in Corsica where pigs roam freely in large areas of maquis shrub land to access natural resources for their diet (Molenat and Casabianca, 1979).

### Humerus 3D models

In this study, we focused on the humerus as this bone is better preserved than other limb bones in archaeological deposits, mainly thanks to their early distal epiphyseal fusion and greater distal density^45^. To compare specimens from the different institutions we used images acquired from medical CT scanners. Specimens from France were CT scanned on a Siemens SOMATOM medical CT scanner with an isotropic spatial resolution ranging from 100 to 500 microns. Specimens from Germany were scanned using the medical CT scanner of the Halle/Saale hospital with the same parameters. For each humerus, 3D surfaces were obtained from the DICOM images stacks Automatic segmentation using the Avizo v 8.0 software (Visualization Sciences Group Inc., Bordeaux). This was carried out using the average value between the maximum density of the bone and the minimum density of the air as a threshold, in order to generate two surfaces corresponding to the external (periosteum) and the internal (endosteum) surfaces of the bone.

### Bone volume

For each specimen, the bone volume of the shaft was defined as the total volume included within the external surface of the bone diaphysis, including the volume of the medulla. Here we have chosen the shaft bone volume instead of the bone length for two reasons. First this approach is meant to be applied to the archaeological record where complete long bones are rare, thus preventing the acquisition of the maximum length. Secondly, bone volume has been proven relevant to discriminate long bones of wild and farmed mustelids^46^. The bone volume was calculated using the ‘Surface Area Volume’ function of the Avizo v 8.0 software. This function calculates the values for the area and volume of the individual patches of a surface (here the diaphysis surface).

### Morphometric mapping of the humeral cortical thickness

**Figure 1:**
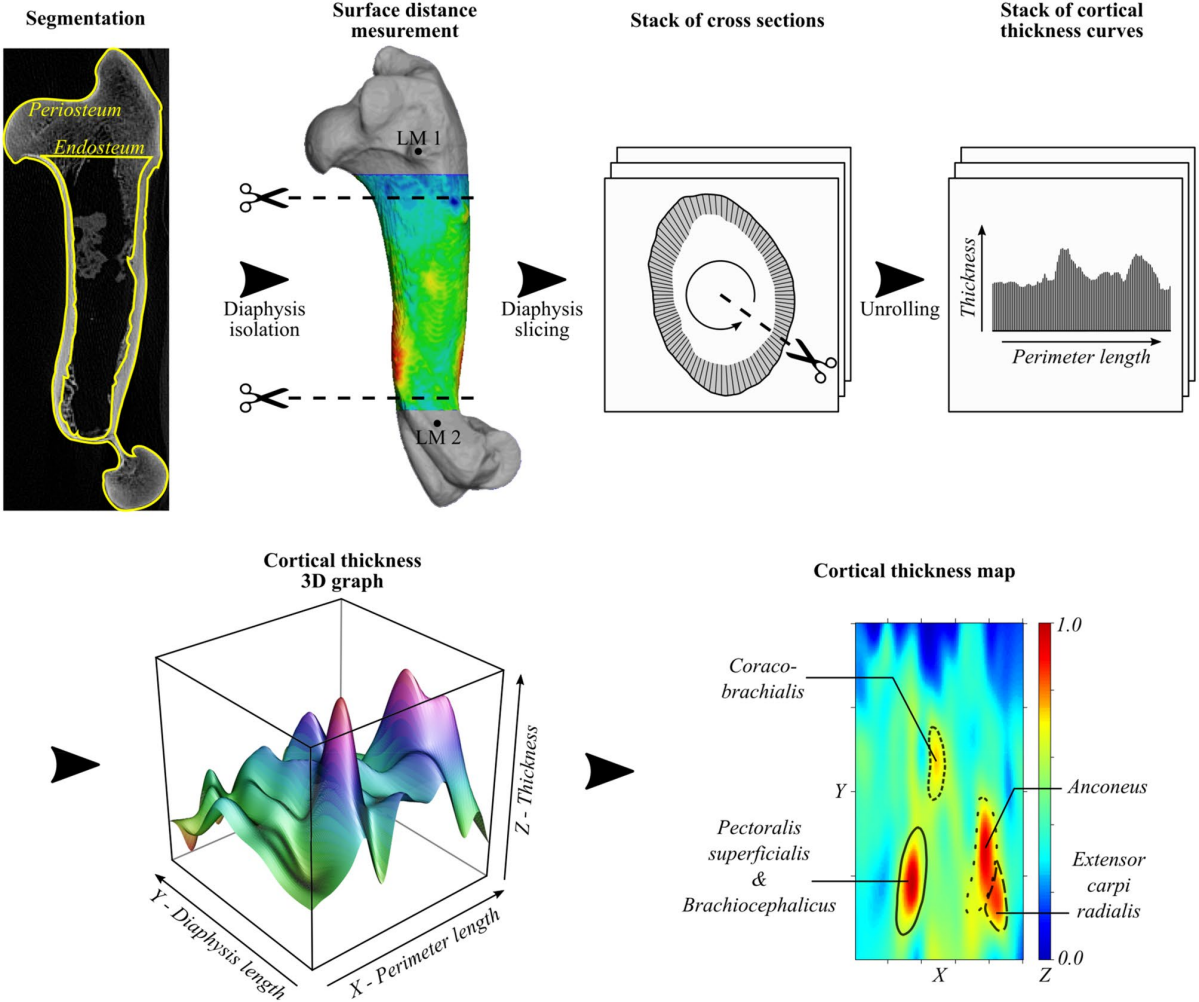
Diagram of the different stages of the Morphometric mapping protocol. The heatmap in the last stage represents the cortical thickness: the minimal distance between the periosteum and endosteum. The greater the thickness the hotter the colour. The circled areas are hand drawn as visual cues and correspond to the attachment sites of muscles according to Barone^47^.

Cortical thickness is defined here as the distance between each point of the periosteum and the closest point of the endosteum^35^. The limits of the analysed portion (i.e., the diaphysis) were defined in two steps (Fig. 1). The first step involved the definition of a controlled cylinder using two planes orthogonal to the longitudinal axis of the diaphysis fixed by two anatomical landmarks near the metaphyses: the extremity of the *teres minor* tuberosity (LM1) for the proximal limit, and the distal fork of the medullary cavity (LM2, internal landmark, visible only in cross section) for the distal limit. The cylinder obtained therefore still had the metaphyses at the end parts due to the position of the landmarks. The second step consisted of removing a margin of 10% on the proximal side and 5% on the distal side to obtain a cylinder corresponding only to the diaphysis.

Given the almost cylindrical shape of the shaft, we used the method A described in^35^ (Fig. 1): the original shape of the shaft periosteum was projected onto a cylinder whose diameter corresponds to the maximum width of the original surface. The cylinder was then cut longitudinally along the cutting line, parallel to the longitudinal axis of the diaphysis which passes through LM1 and is unrolled on a plane. The direction of the unrolling is processed according to the laterality of the bone, ensuring completely comparable maps irrespective of the laterality^35^. The unrolling method slightly adjust the shape of the diaphysis (that is not a perfect cylinder) to make it fit into a rectangle during the unrolling process. With the standardization, the absolute height and circumference of the diaphysis cannot be directly represented by the height and width of the maps. The maps approximate the unrolled diaphysis shape, but they are not directly equivalent.

### Standardization

To statistically compare the topographic variation in cortical thickness, it was necessary to standardize the measurements and to map the cortical thickness of the shaft. This second aspect is critical, since any comparison should ideally be based on homologous landmarks of the original 3D object. Because there were few or no truly homologous landmarks on these cylindrical regions, the solution adopted was to map the original surface by means of a regular mesh of K rows and M columns. Here, M = 100 and K = 200 in order to maximize the spatial resolution of the grid without unduly increasing the computation time and to maintain a square cell shape. Using this method, the values of cortical thickness at the grid intersections were evaluated by thin plate spline regression of the original data^48^. Before the thin plate spline regression, the thickness values were standardized between 0 and 1. The resulting mapping represents the standardized thickness estimated at the intersection of the same number of grid lines projected on the exterior surface of each original shape. The use of a flat thin plate spline regression, which is part of a statistical approach based on generalized additive modelling (GAM)^49^, facilitates statistical comparisons. After the GAM, we obtained a table of M = 100 columns and K = 200 rows containing the cortical thickness values ranging between 0 and 1. For each cell the column number was given the value of x (diaphysis length), the row number the value of y (diaphyseal circumference), and the cortical thickness the value of z, we therefore obtained a new table of 20,000 rows and 3 columns (x, y and z) which can be analysed statistically (Fig. 1). Another property of the GAM-based approach was the ability to construct consensus maps using many specimens from a sample by merging all of the individual information into a single dataset and evaluating the effectiveness of consensus using generalized cross-validation (GCV)^49^.

### Life history dataset

To test potential covariations between life-history traits and humeral cortical thickness in wild boar we collected body mass, sex, and age for the specimens (See data availability). Captive-raised wild boars have a known age of death but wild-caught wild boars had to be aged according to their dental eruption and occlusal attrition stages^50,51^.

### Muscle force estimates

To measure the covariation between the humerus cortical thickness and the functional properties of the muscles attached to the humerus we dissected the *anconeus* (ANC), which abducts on the ulna and allows an extension of the elbow; the *brachiocephalicus* (BRA), which originates at the neck and the back of the head and allows the protraction of the humerus; the *coracobrachialis* (COR), which originates on the coracoid process of the scapula and allows retraction of the humerus and adduction of the arm; the *extensor carpi radialis* (ECR), which inserts on the metacarpal tuberosity and allows an extension of the carpal joint and flexion the elbow joint; and the *pectoralis superficialis* (PEC), which originates on the sternum and allows the adduction and retraction of the limb^47^, of 22 experimental captive-reared wild boars, one specimen from the control population and five wild boars from French populations (Table 1, See data availability). Muscle data were not available for other specimens. The muscles were weighed to the nearest gram and we measured muscle fascicle length with callipers. Based on the known density of mammalian muscle (1.06 g/cm^−3^) (Mendez et al., 1960) we calculated the anatomical cross-sectional area (ACSA) as a proxy for muscle force^52^.

### Statistical analyses

#### Size, shape and life trait covariations in wild-caught and captive wild boars

The difference in humerus volume and body mass variation among captive-reared and wild-caught wild boars, taking into account their sex, was visualized with a box plot and tested with factorial analysis of variance (ANOVA). The correlation between humerus volume and body mass and between humerus volume and age, taking into account sex, was tested using the Pearson correlation test. The cortical thickness difference among wild-caught and captive-reared wild boars while accounting for age, body mass, and bone volume was explored using a factorial MANCOVA with 1000 permutations.

We tested and visualized the ACSA differences among wild-caught and captive-reared wild boars using ANOVA and a box plot. To visualize and test the covariation between body mass, bone volume, age, and intrinsic muscle force with topographic bone variation of the cortical across the shaft in wild-caught and captive wild boars, we used a two-block partial least squares (2B-PLS) analyses^53,54^ using the standardized morphometric maps as variables.

#### Comparing plastic size and shape response to captivity with changes induced by artificial selection

Bone volume differences between wild boars and domestic pig groups were tested using an ANOVA with the pairwise comparison tests (Bonferroni correction) and visualized with a box plot. The cortical thickness differences were tested with a Procrustes ANOVA and visualized using a Canonical Variate Analysis (CVA); both were performed on a cortical thickness reduced dataset after a Principal Component Analysis (PCA) performed on the Procrustes coordinates to keep 95% of the variance^55^. To visualize cortical thickness variations along the canonical axes we calculated the theoretical minimum and maximum morphometric map for each axis. On each cartography, attachment sites of the four muscles are displayed as visual cues. We also visualized the consensus map of wild-caught wild boars, captive-reared wild boars, captive-domestic pigs and free-ranging domestic pigs to facilitate the interpretations. Deformation maps between two consensus maps were calculated by subtracting the values of the consensus map. To differentiate the topographic variation of the cortical thickness between wild boars, traditional pig breeds and improved pig breeds, taking into account their genetic history and their sex, we performed a factorial MANOVA with a 1000 permutations procedure.

All the statistics were performed using R (R Core Team. 2017). Factorial MANOVA, Procrustes ANOVA and PLS were performed using the R package "geomorph"^56^. ANOVA, PCA were performed using the package “stats” (R Core Team. 2017), CVA using the package “Morpho”^57^ and visualizations were performed by using “geomorph”.

## Results

### Lifetime changes in humerus cortical thickness in wild boars associated with mobility reduction

**Figure 2:**
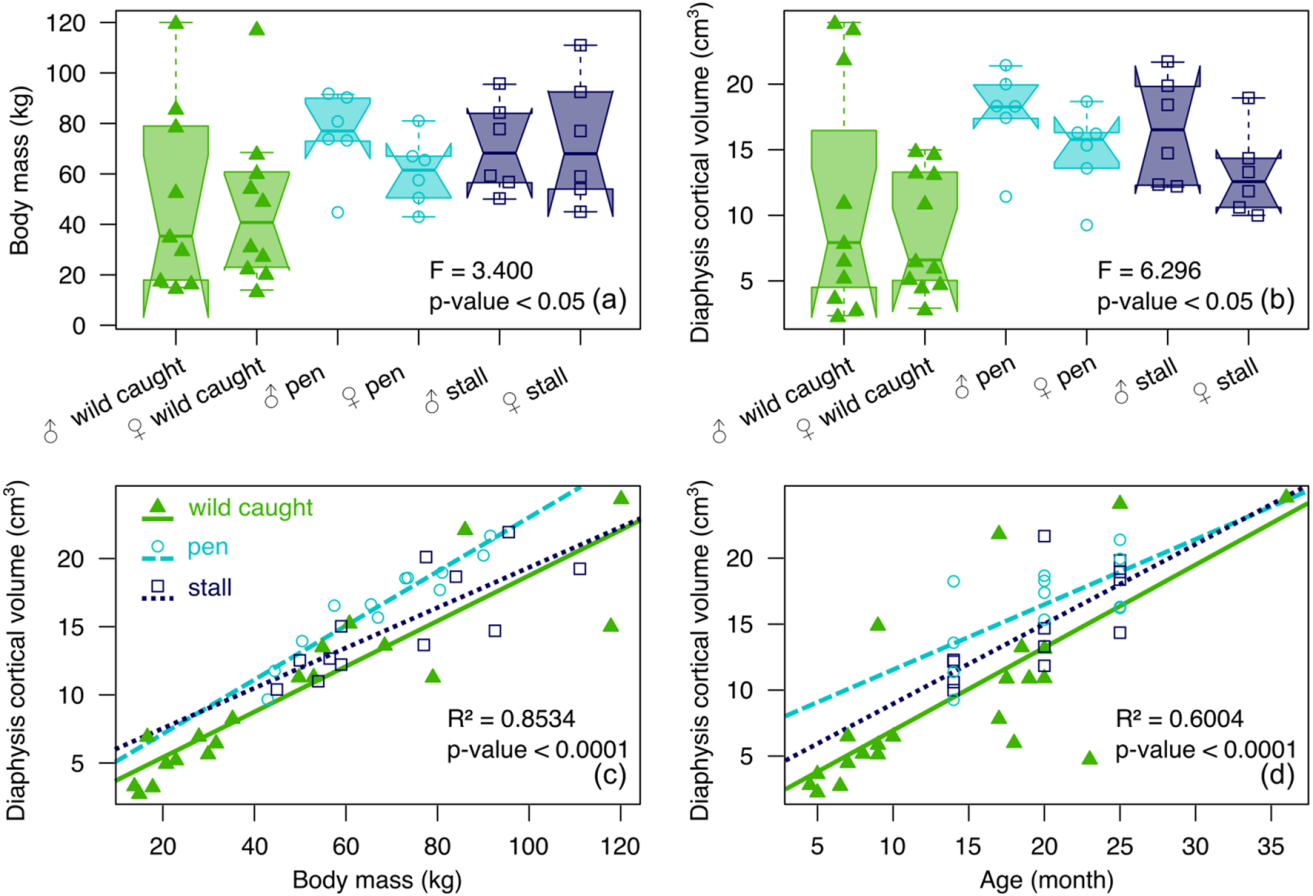
Box plots displaying differences among captive-reared and wild-caught wild boars in (**a**) body mass and (**b**) bone volume. The box represents 50 percent of data (interquartile) and the horizontal bar inside is the Median. The “notch” represents 95% confidence interval of the Median. The lower and upper whiskers represent respectively the minimum and maximum values. Regressions between (**c**) bone volume and body mass, and (**d**) bone volume and age, among wild-caught and captive-reared wild boars.

Free-ranging wild boars did not differ in body mass compared to pen (*P* = 0.149) and stall (*P* = 0.064) boars (Fig. 2 a) even when we comparing wild-caught and captive wild boars using the same age range (pen: *P* = 0.18; stall: *P* = 0.71). We also found that compared to wild caught wild boars, captive specimens have greater bone volume either in pen (*P* < 0.01) or in stall (*P* < 0.01) (Fig. 2b). In addition, males wild boars had a higher bone volume than females (*P* < 0.05) (Fig. 2b). The correlation between bone volume and body mass is strong for free-ranging boars (slope: 0.20, *P* < 0.0001, R^2^ = 0.89) and those raised in the pen (slope: 0.20, *P* < 0.0001, R^2^ = 0.89), but weaker for wild boars raised in a stall (slope: 0.15, *P* < 0.01, R^2^ = 0.61) (Fig. 2c). The correlation between bone volume and age is strong for free-ranging wild boars (slope: 0.63, *P* < 0.0001, R^2^ = 0.54), but weaker for pen boars (slope: 0.60, *P* < 0.01, R^2^ = 0.48) and stall boars (slope: 0.49, *P* < 0.05, R^2^ = 0.38) (Fig. 2d).

**Figure 3:**
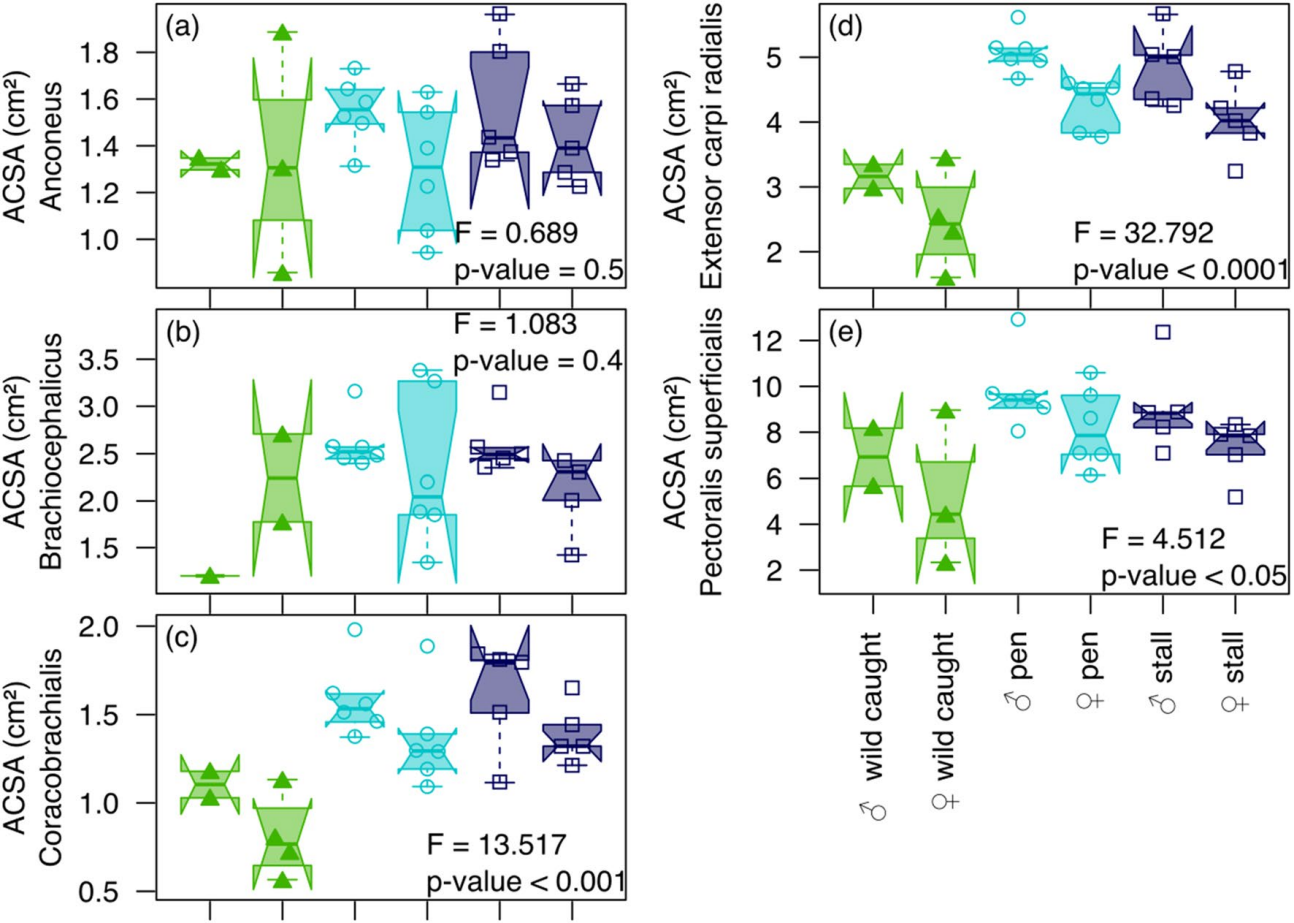
Box plots displaying differences in muscle ACSA among wild-caught and captive-reared wild boars taking into account their sex for (**a**) the anconeus, (**b**) the brachiocephalicus, (**c**) the coracobrachialis, (**d**) the extensor carpi radialis, and (**e**) the pectoralis superficialis. The box represents the 50 percent of data (interquartile) and the horizontal bar inside is the Median. The “notch” represents the 95% confidence interval of the Median. The lower and upper whiskers represent respectively the minimum and maximum values.

Captive boars generally have higher ACSA values (Fig. 3a–e) than free-ranging boars, especially for the extensor *carpi radialis* (Fig. 3d, F = 32.79, *P* < 0.0001), the *coracobrachialis* (Fig. 3c, F = 13.52, *P* < 0.001), and the *pectoralis* (Fig. 3e, F = 4.51, *P* < 0.05). Moreover, males have higher ACSA values than females, especially for the extensor *carpi radialis* (Fig. 3d, F = 17.04, *P* < 0.001), the *coracobrachialis* (Fig. 3c, F = 6.61, *P* < 0.05), and the *pectoralis* (Fig. 3e, F = 5.14, *P* < 0.05).

**Figure 4:**
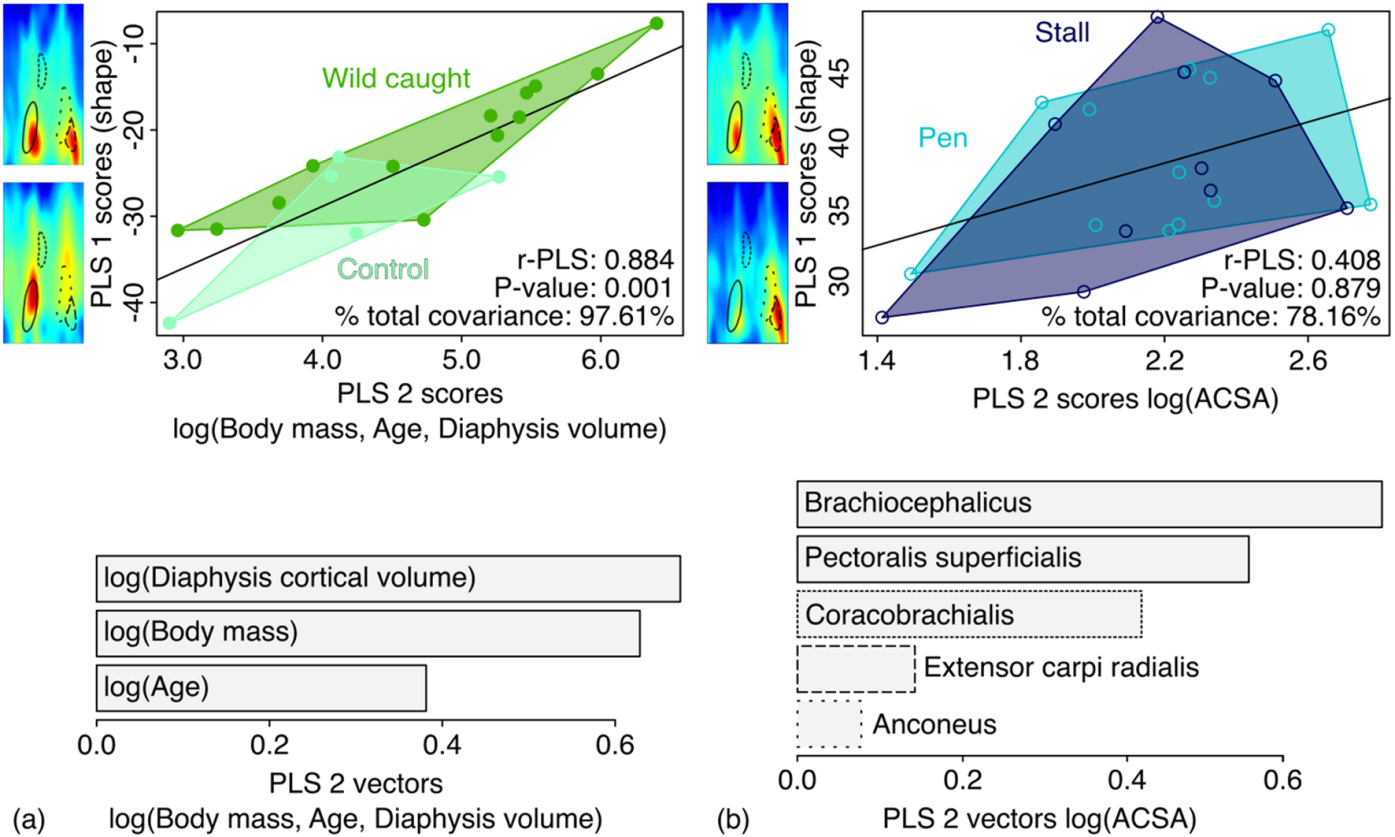
PLS regression between the humerus shaft cortical topography block and (**a**) life history traits (body mass, age, bone volume) and (**b**) muscle ACSA blocks. Wild-caught wild boars are visualized in the filled green circles and captive-reared wild boars in the open blue circles. Black lines represent the PLS regression line. Shaft cortical topography deformations are visualized with two extreme cortical thickness maps where muscle attachment sites are encircled as visual cues. Singular vectors are shown using a barplot for life history (**a**) muscle ACSA and (**b**) blocks.

The covariation between the cortical topography of the humerus in free-ranging wild boars and body mass, age and bone volume (Fig. 4a) is highly significant. We can see on the visualizations that an increase in body mass, age, and bone volume induce the shift of muscular attachment towards the distal part of the bone. The contrast in thickness between the different zones also increases. In addition, the relative thickness of the muscle attachment area of the ECR (bottom right) increases sharply with this increase in body mass, age, and bone volume. The three parameters, mass, age and volume have a comparable impact on the variations in cortical thickness (Fig. 4a), even if the effect of age is slightly less important.

On the other hand, there is no significant covariation between the cortical topography of the captive boars and the ACSA of the limb muscles (Fig. 4b).

### Relative impacts of locomotor behaviour and selection in humerus cortical thickness

**Figure 5:**
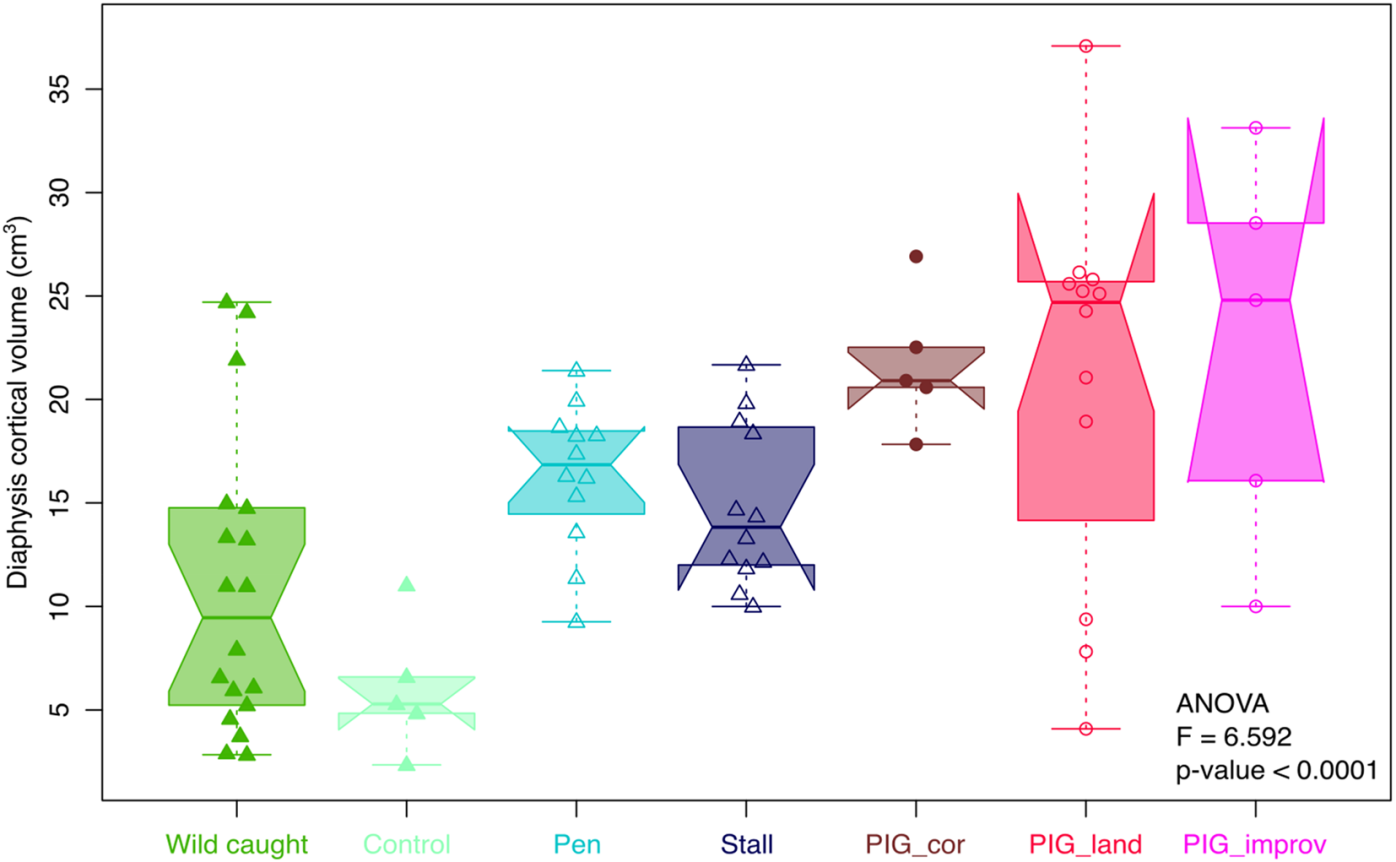
Box plots displaying humerus bone volume variation in wild boars (triangles) and pigs (circles). Free ranging (filled) or captive environments (open) are also indicated. The box represents the 50 percent of data (interquartile) and the horizontal bar inside is the Median. The “notch” represents the 95% confidence interval of the Median. The lower and upper whiskers represent respectively the minimum and maximum values.

Bone volume varied significantly among wild boars (wild caught and captive) and pigs (ANOVA: F = 9.115, *P* < 0.001). Pigs have significantly higher bone volume than wild boars but captive boars have an intermediated range of humerus bone volume, greater than wild-caught wild boars but lower than free-ranging and improved pig breeds (Fig. 5).

**Figure 6:**
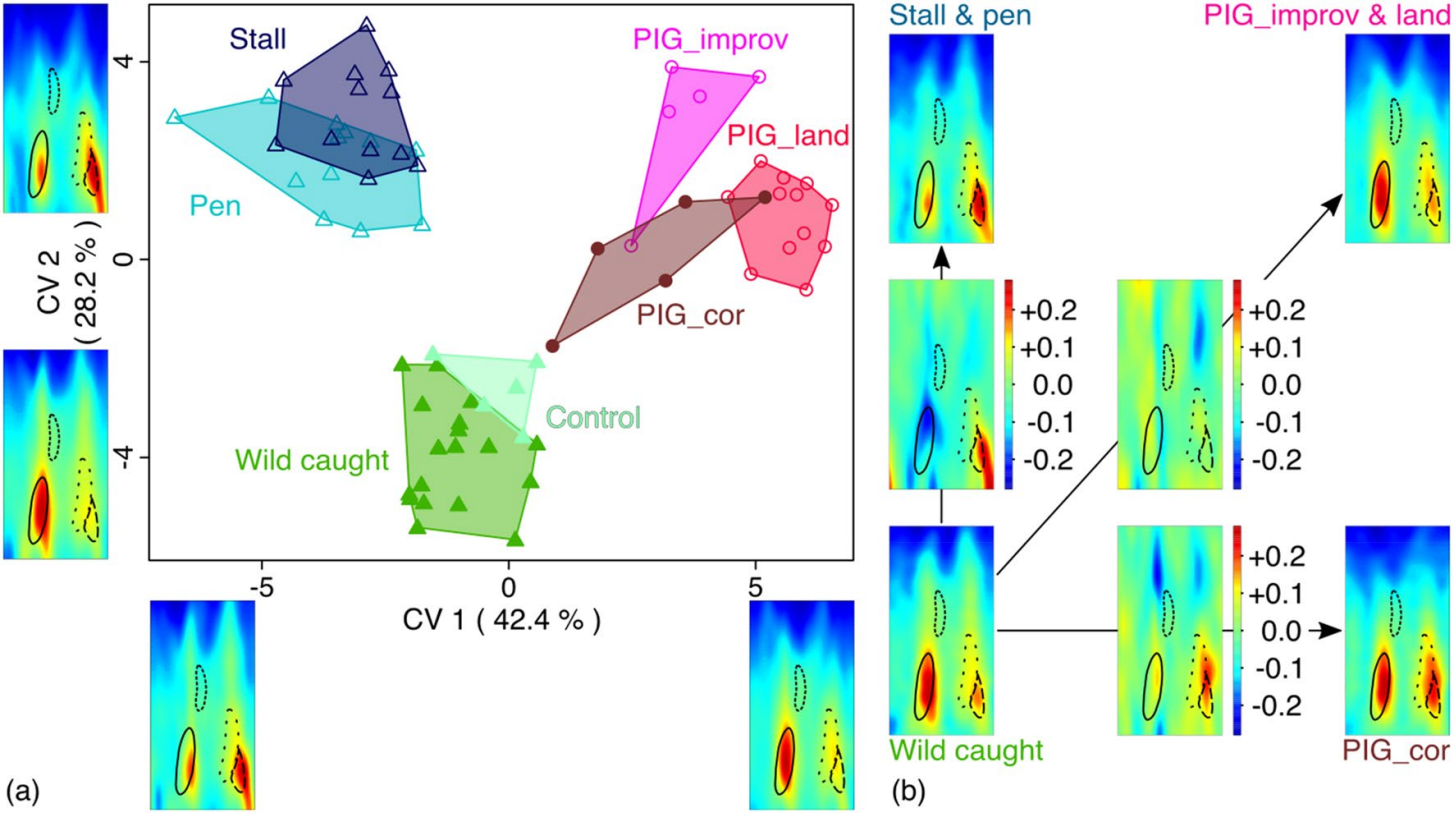
(**a**) Morphospace based on a CVA representing the pattern of humerus shaft cortical topography divergence among wild boars and pigs living in free-ranging or captive environments. Minimum and maximum shaft cortical topography are shown for each axis. See Fig. 1 for the identification of each muscle attachment in the consensus map. (**b**) Consensus maps are shown for four groups: wild-caught wild boars (WB_wc & ctrl; GCV = 0.009016), captive-reared wild boars (WB_stall & pen; GCV = 0.006548), captive pigs (DP_improv & land; GCV = 0.011224), and free-ranging pigs (DP_cor; GCV = 0.003657). Each consensus map is compared with the wild-caught wild boars map, and the deformation map is displayed between them. On each map, attachment sites of the four muscles are displayed as visual cues.

Cortical thickness topography varies significantly among wild boars (wild caught and captive) and pigs of our dataset (MANOVA: F = 2.6156; *P* < 0.0001). The factorial MANOVA shows that significant differences can be observed between wild boar populations and pig breeds (traditional, industrial) (R^2^ = 0.048, *P* < 0.05) and between sexes (R^2^ = 0.016, *P* = 0.345). The interaction denotes that sexual dimorphism differs among the wild and domestic populations (R^2^ = 0.011, *P* = 0.512) but with almost no influence on the total variance. The first axis 1 of the CVA displays the divergence between the captive boars and the pigs respectively in the negative and the positive side of the shape space (Fig. 6a). The difference in cortical thickness topography between captive boars and pigs corresponds to a relative change in the muscle topography between the area of the PEC and BRA entheses on the one hand and the area of ECR enthesis on the other. The two areas of muscular insertion are relatively thick in captive boars while in pigs, only the PEC and BRA area show a relative increase in thickness. The CV2 distinguishes free-ranging boars in the negative part from captive boars and pigs in the positive part. Captive boars are also distinguished from their free-ranging counterparts on this axis, but animals raised under the two contexts of captivity (enclosure and stable) have a very similar cortical thickness. For pigs, free-ranging Corsican pigs seem to have a cortical thickness that is intermediate between captive pigs and free-ranging boars.

The comparison of the consensus cortical topography of the four main morphological groups (Fig. 6b) shows that the difference between free-ranging and captive boars mainly corresponds to a sharp relative decrease in cortical thickness at the attachment site of PEC and BRA, as well as a relative increase in cortical thickness at the ECR attachment site. The difference between free-ranging boars and free-ranging pigs corresponds to a relative increase in cortical thickness at the attachment sites of ANC, ECR, PEC and BRA and a decrease in two zones in the proximal part of the shaft. At these muscle attachment sites, the difference between free-ranging boars and captive pigs is moderate, resulting in a slight overall increase in thickness associated with a slight thickness decrease at in the proximal part of the diaphysis.

## Discussion

In this paper we tested whether the cortical structure of the humerus could record changes in the locomotor behaviour of a wild ungulate during growth due to captivity. Understanding these effects is key to documenting the early process of animal domestication in archaeology prior to genetic isolation and breeding selection. However, quantifying a functional signal in long bone diaphyses allowing for the reconstruction of past behaviour from long bone morphology has so far been challenging^37,38,58^. Our project has exploited the results of a unique experiment where locomotor effects can be explored at the scale of wild ungulate populations while controlling for genetic and environmental factors. In association with a comprehensive morphometric mapping of the humerus shaft, this study provides evidence that the functional changes associated with captivity are recorded in the internal structure of limb bones, thus offering a new methodological perspective to understand the early stages of animal domestication.

This experiment confirms that the cortical bone volume of the humeral shaft in free-ranging populations of wild boars is strongly influenced by the interrelated effects of body weight increase, growth and sexual dimorphism^37,59,60^. This experiment shows that mobility reduction increases the bone volume of the shaft in captive wild boars compared to their wild counterparts when sex differences are taken into account, supporting previous studies showing that captivity increase the body weight and accelerates growth in many mammalian taxa when the diet is nutritiously balanced^46,61,62^. However, exercise during growth is known to stimulate bone modelling^20^ and a reduction of cortical thickness has been observed in the jaw bones of captive weeper capuchin^63^. Therefore we expected that captive wild boars, with less frequent activities and a lower motion intensity, would have displayed less cortical volume than free-ranging individuals. However, we observed that mobility reduction increased the weight and age-related bone volume. This suggests that developmental disruptions through the body mass increase in captivity could have changed the biomechanical loads during puberty. Despite a reduction of mobility, body mass increase in captivity could have driven bone growth, providing some resolution to this paradox. This bone robusticity associated with a body mass increase has a correlate in humans. Indeed, obesity has been shown to increase the bone density in men and women through the stimulation of bone formation induced by the mechanical loading conferred by weight^64,65^.

This experiment provides further evidence that growth in captivity impacts the muscular system of wild boars, with captive specimens displaying greater muscle cross-sectional areas. Although presumably less physically active than their wild counterparts, captive wild boars did not display a localized increased of the scapular bracing muscular apparatus shown in the entheseal change of captive reindeer^66^. Here, we found an overall increase in the cross-sectional area of the humerus muscles which was previously also demonstrated for muscles attached to the calcaneum^67^ and described for other mammals bred in captivity^61^. Until further ethological and physiological studies in captivity have been performed, we can only hypothesize that this increase in the muscular system of captive specimens is the cumulative and interrelated consequences of (1) increased body mass, (2) protein rich diet, and (3) stereotypical behaviour increasing the frequency of muscular use. All these factors are likely to be responsible for reinforced muscle attachments. Our experimental specimens were fed nutritionally balanced pellets (15% protein) dedicated to pig farming to ensure steady growth and bone formation^59^, which may have provided the opportunity for muscle growth beyond what is possible under natural conditions.

Our experimental study showed that the topographic variation in cortical thickness of the humeral shaft is multifactorial and strongly influenced by age and body mass. Our results further show that body mass and its associated bone volume increase are key to predicting cortical thickness variation, in contrast to previous studies on primates^37,59,68–70^; although differences in the functional use of the forelimb between wild boars and primates could explain this inconsistency. We did not detect significant covariation between the muscular cross sectional-areas and the relative topography of the cortical thickness of the humerus in wild boars. These results are congruent with the hypothesis that in vivo muscular load does not affect entheses unless they are pathological^42^. We strongly suspect, however, that covariations were simply not detected, as the impact of the muscle-cross sectional area on the cortical thickness topography is likely reflected in absolute rather than relative thickness differences. As thickness values were normalized on a 0–1 scale before the GAM standardization, this only allowed the detection of relative thickness differences along the shaft.

We found that mobility reduction significantly impacted cortical thickness topography, supporting the hypothesis that captivity can be inferred from bone structure, a signal already acknowledged in studies on primates^37^ and reindeers^66^. Captive and free-ranging wild boars from the same population and of the same age showed distinct patterns of cortical thickness at the distal part of diaphysis. Compared to the free-ranging wild boars, captive individuals showed a distinct relative reduction of the cortical thickness located at the *brachiocephalicus* and *pectoralis superficialis* entheses and a relative increase of the *extensor carpi radialis,* suggesting decreased use of the extensors of the forelimb in captive specimens. Changes in cumulative use instead of load can probably better explain how muscular activity might impact the diaphyseal cortical thickness.

Selection due to domestication has also left a clear signal on the volume and topography of the cortical bone of the humerus. The selective breeding of the last 200 years drastically increased the humerus cortical volume, with current pig breeds showing an almost two-fold increase of the cortical bone volume compared to extant wild boars from France. The selective breeding of pigs has also impacted the humerus cortical thickness topography along a very different functional trajectory than what is observed due to mobility reduction. Compared to free-ranging and captive wild boars, pig breeds display an increase of the *anconeus*, an extensor of the forelimb. This phenotypic divergence could be related to breeding selection^71^ inducing multifactorial and interrelated influences. Selection on early growth probably had a drastic impact on the developmental programming of the diaphysis bone morphology. Moreover, selection for large muscles and quick gain of body weight potentially impacted the gait of the animal and may have affected cortical bone volume and its topography. This divergent trajectory in cortical topography between wild boars which cannot fully express their locomotor behaviour and pigs from traditional or industrial breeds selected for meat production, suggests that the anthropogenic control of a movement in a wild ungulate and the selective breeding for body growth induce different responses in the cortical morphology. Therefore, both anthropogenic influences over ontogeny of an ungulate should be possible to discriminate in the archaeological record.

## Conclusion

We demonstrated that despite the multifactorial influences of ontogeny, sex, and body mass on the humerus cortical shaft volume and topography, changes in locomotor behaviour induced by captivity produced changes in the cortical topography beyond what is observed in the natural habitat and along a different phenotypic trajectory than changes induced by recent selective breeding. These results provide an experimental proof of concept that changes in locomotor behaviour and selective breeding might be inferred from long bones in the archaeological record. However, this proof of concept now needs to be confronted to the archaeological records of *Sus scrofa* since the early Holocene to assess whether the trends in the cortical thickness observed under experimental constraints and with modern populations could prove relevant to explore the process of domestication and the role of anthropogenic forces in the evolutionary changes of wild species^72^. This survey should include contexts of hunter-gatherers societies before the Neolithisation and performed across the contexts of emergence of animal domestication until at least periods when the domestication syndrome is clearly observable in the archaeological record.

Further studies are now required to explore the impact of mobility reduction in the trabecular architecture of the humerus and the developmental changes behind these plastic responses. Large scale muscular measurements from wild populations are also required to build biomechanical models^73^ needed to fully infer the functional changes induced by captivity on limb bone morphology.

## Data Availability

Code, data and metadata to perform statistical analyses are archived in the Dryad Digital Repository (https://datadryad.org/) and are currently accessible at the following location: https://datadryad.org/stash/share/q04zdMbnHJwY0EciL_DqDwKtfVafkAOTvFvbLu5SG7g.
